# Expression and role of p16 and GLUT1 in malignant diseases and lung cancer: A review

**DOI:** 10.1111/1759-7714.13651

**Published:** 2020-09-18

**Authors:** Aldo Pezzuto, Michela D'Ascanio, Alberto Ricci, Alessandra Pagliuca, Elisabetta Carico

**Affiliations:** ^1^ Cardiovascular‐Pulmonary Science Department Sant' Andrea Hospital‐Sapienza University Rome Italy; ^2^ Clinical and Molecular Medicine Department Sant' Andrea Hospital‐ Sapienza University Rome Italy

**Keywords:** Glucose transporters, lung cancer, p16 expression

## Abstract

Non‐small cell lung cancer (NSCLC) is the leading cause of cancer death and in most cases it is often diagnosed at an advanced stage. Many genetic and microenvironmental factors are able to modify the cell cycle inducing carcinogenesis and tumor growth. Among the metabolic and genetic factors that come into play in carcinogenesis and tumor cell differentiation and growth there are two different proteins that should be considered which are glucose transporters (GLUTs) and p16^INK4^ The first are glucose transporters which are strongly involved in tumor metabolism, notably accelerating cancer cell metabolism both in aerobic and anaerobic conditions. There are different subtypes of GLUT family factors of which GLUT 1 is the most important and widely expressed. By contrast, p16 is mainly a tumor‐suppressor protein that acts on cyclin‐dependent kinase favoring cell cycle arrest in the G1 phase. Our search focused on the action of the aforementioned factors.

## Graphical abstract

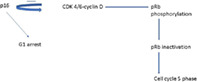

This review highlights the role of p16 and GLUT‐1 and GLUTs proteins in malignancies especially in lung cancer, focusing on the pathways activated leading to cell cycle dysregulation, cancer growth and migration. Since they have a clinical prognostic value, they may become targets for novel therapies.

## Introduction

Lung cancer is the leading cause of cancer‐related death.^1^ Adenocarcinoma is the most common histotype, followed by squamous cell carcinoma and large cell carcinoma.^2^ The broadest classification of lung cancer is in small and non‐small cell cancer (NSCLC) which is the prevalent subtype.

Cigarette smoking is by far the main avoidable cause of both NSCLC and small cell lung cancer (SCLC) often preceded by lung nodule detection.^3^, ^4^, ^5^ It is estimated that the prevalence of NSCLC will continue to rise, partially due to the rise in tobacco use and also to an increase in air pollution.^6^

NSCLC is usually symptomatic and is therefore diagnosed at an advanced stage. Despite recent advancement in therapies, the prognosis of NSCLC patients remains unsatisfactory with a five‐ or 10‐year survival rate of 15%–20% of the total population without *EGFR* gene mutation or ALK translocation.^7^

Over the last decades many screening programs and new techniques for early detection have been developed to improve the prognosis.^8^ Some criteria have been identified as being associated with a high risk of patients developing lung cancer such as a smoking habit of a pack‐year of more than 20, family history of cancer, characteristics and location or shape of lung nodules.^9^

New prognostic and target molecules need to be determined to predict lung cancer development and evolution.

Our study focused on two different proteins and their associated genes both involved in a different manner but at the same time resulting in lung carcinogenesis and tumor growth.

Glucose transporters (GLUTs) are a family of proteins that favor the transport of glucose in the blood. Several members of glucose transporters have been identified from GLUT‐1 to GLUT‐12.^10^

GLUT proteins have both an intracellular and transmembrane domain. Several conditions and factors are able to stimulate the aforementioned, such as hypoxia and inflammatory factors such as IL‐6, and VEGF.^10^

**Figure 1:**
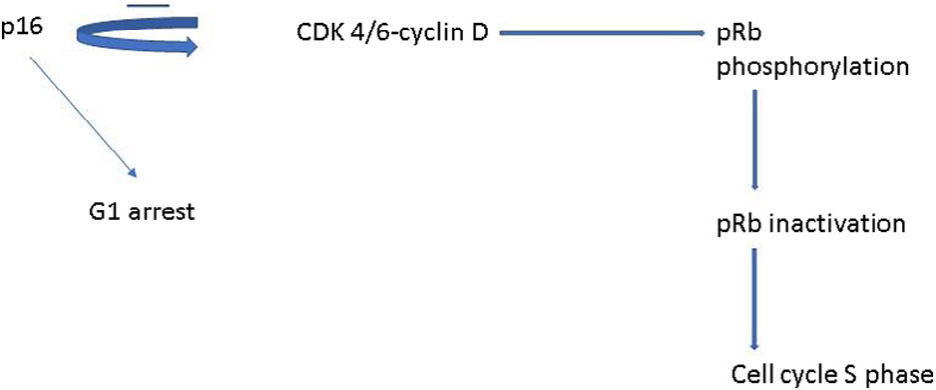
p16 is able to inhibit the action of cyclin D kinase which is responsible for retinoblastoma protein (pRb) phosphorylation leading to pRb inactivation and cell cycle progression stimulating the S phase. By contrast, p16 action leads to G1 arrest of the cell cycle.

p16 protein is an antioncogene, recognized as a tumor suppressor whose action takes place by inhibiting the phosphorylation of the retinoblastoma protein (pRb) by binding to the cyclin‐dependent kinase complexes (CDK4–CDK6) which leads to arrest in phase G1 of the cell cycle (Fig 1).^11^

## Methods

A literature search was carried out using the main databases such as Pubmed, Google Scholar, and Scopus with lung cancer pathways, GLUTs and p16 as keywords. We also included some criteria found in English language papers published by peer reviewed journals, as well as studies carried out in humans, in vitro and in vivo focusing on the expression of GLUT and p16 in metastatic malignant cancers. Clinical trials or cohort studies were included if the considered endpoint was survival with a sample size of at least 50 patients. Studies concerning patients with severe comorbidities were excluded.

The differences in GLUT and p16 expression are reported in this review.

## Results

### p16

Among the proteins and factors involved in carcinogenesis and tumor cell proliferation the role of p16 should be taken into account.^11^ The locus where p16 gene is included is located on human chromosome 9p21 and is frequently subjected to deletions hindering its action as a tumor suppressor. Its main function is in cell cycle regulation inhibiting the pRb pathway by downregulation of cyclin‐dependent kinases.

#### p16 in human studies

p16 protein and its related gene are associated with HPV infection and its activity has been studied in different cancers including cervical cancer.^12^ The p16 positive expression associated with HPV infection has been studied as a predictive factor in different cancers such as head and neck cancer, showing its association with a better response after radiotherapy.^13^ Another type of cancer in which p16 plays a role is oropharyngeal cancer; indeed, it has been found to be a prognostic factor associated with a better outcome and a decreased epidermal growth factor (EGFR) expression.^14^ in vitro studies have confirmed that there is a relationship between p16 and EGFR activation.^15^ Expression of p16 protein has also been found to affect patient survival in tonsillar carcinoma.^16^, ^17^

Investigating its action, p16 inhibitor kinase 4 (p16‐INK4a) protein has a negative regulatory role in the cycle of eukaryote cells by inducing their arrest during the course of differentiating processes.^18^ Conversely, p16 gene inhibition leads to phosphorylation of pRb, unblocking the cell cycle with a subsequent uncontrolled cell growth and increased proliferation in all cancer types.^19^ Several genetic alterations can induce inactivation of p16 including homozygous deletions, promoter hypermethylation, point mutations and loss of heterozygosity (LOH).^20^ Homozygous deletions and promoter hypermethylation lead to its dysregulation, whereas point mutations and small deletions, in particular misssense mutation, alter the structure and activity of p16.^21^ Thus, p16 deregulation is favored by different transcriptional factors and oncogenes. Genetic inactivation of p16 may be compensated by the activation of other antioncogenes such as p21. This suggests that carcinogenesis is the result of an imbalance between oncogenes and oncoprotein activation and their inhibition.

There are different subtypes of inhibitors of cyclin‐dependent kinases such as p16INK4a, p15INK4b, p18INK4c, all of which are able to affect the cell cycle.^22^ There is a cross‐talk among p16INK and transcription factors such as NF‐kB which induces cell growth and cancer proliferation. p16 is able to compete with NF‐kB to bond with cyclin‐kinases.

Previous studies have demonstrated that p16‐INK4a is overexpressed in cancers and acts as a tumor suppressor. It is closely associated with cell death and tumor aggressiveness.^23^ The downregulation of p16 leads to cancer progression but overexpression is associated with poor prognosis in different solid cancers.

#### Preclinical studies

The p16 gene is CDKN2A which encodes two different genes ‐ p16 INK4a gene and p14ARF ‐ which in turn is involved in apoptosis.^24^ Their common function is to inhibit cell cycle progression. Some amino acid residues of p16 have been identified that interact with cyclin D‐dependent kinases cdk4 and cdk6 inhibiting pRb phosphorylation.^25^

An antiapoptotic role played by p16 has been shown to be associated with downregulation of proapoptotic proteins such as Bax‐Bak and cell cycle regulator Bcl‐2, and sensitization of proliferating cancer cells to cisplatin activity.^26^ The latter is an antineoplastic agent which acts by alkylating cancer cell DNA, and its action is sensitive to different agents. The involvement of Bax in the apoptotic process counterbalances the action of procarcinogenic molecules. In Table 1 the main factors activated with the associated effects are represented and Fig 1 depicts the main pathway.

**Table 1:** Effects of p16

Factors involved	Pathway
pRb hypophosphorylation	Inhibition CDK4 and CDK6 and G0 arrest
P21 activation	Cell cycle arrest
P53 coactivation	G2 cell cycle arrest
P15‐INK4B coactivation	Tumor‐suppressor

### p16 in lung cancer

p16 tumor suppressor undergoes mutational and epigenetic alterations in several human cancers and acts as a critical target for the inactivation of several DNA tumor viruses.

#### Preclinical studies

A potential role for p16 has been found in early lung cancer detection and preneoplastic lesion evaluation. It has been found to be useful in the discrimination between adenoma and adenocarcinoma when associated with heterogeneous nuclear ribonucleoprotein such as hnRNP, cyclin D1 and ki67.^26^ Overexpression of cyclin D1 and decrease of p16 have been reported to be frequently observed in both adenocarcinoma and adenomatous hyperplasia.^26^, ^27^

The interaction of p16/pRb as oncosuppressor genes has been found to be correlated with telomerase activity in adenocarcinoma of the lung in adenocarcinoma cell lines.^28^ The relationship between p16 and activating mutation markers could influence the stepwise progression of tumor cells, inhibiting the mammalian target of rapamicin (mTOR) pathway and downstream activation of hypoxia factor.^29^

p16 as a tumor suppressor gene has been shown to interact with P53 increasing the action of cell cycle regulation.^30^ The observation that p16 gene preferentially undergoes gene silencing through promoter hypermethylation is of crucial importance in the evidence of protein expression in NSCLC.^31^

#### Studies in humans

The protein expression level in surgically‐treated NSCLC has been reported to be associated with a poor prognosis probably due to a compensation to its inactivation by tumor factors.^32^

p16‐INK as well as p40 and p63 has been found to be overexpressed not only in malignant but also in benign lung adenoma.^33^

Among the malignant lung diseases, abnormal expression of p16 has been found mainly in NSCLC in which methylation of p16 gene is frequently observed.^34^, ^35^, ^36^ Hypermethylation of the p16 gene together with p53 and *KRAS* mutation has been reported to promote lung carcinogenesis in smokers.^37^, ^38^, ^39^

The p16 protein has been associated with the X‐linked inhibitor of apoptosis protein (XIAP) in both benign and malignant neoplasm such as adenoma, and bronchiolo‐alveolar carcinoma of the lung.^40^, ^41^ The X‐linked inhibitor of apoptosis has, in turn, a prognostic role in NSCLC and has a relationship with antiapoptotic molecules such as caspase. The overexpression of XIAP is able to revert the action of caspase. Thus there is a network of genetic and transcriptional factors that cross‐talk and influence cell growth and death.

## GLUT protein

### Preclinical studies

Among the GLUT isoforms there is a significant elevation in the level of GLUT‐3 and GLUT‐5 mRNAs in tumor cells, notably in hypoxic conditions, meaning that cancer cells increase their glucose intake and have higher metabolism rates. These isoforms are differently but ubiquitously distributed in several tissues. The rate of glucose transport depends upon changes in glucose gradient as demonstrated in in vitro and in vivo experiments.^42^, ^43^

Elevated GLUT1 expression has been previously described in many cancers, including hepatic, pancreatic, breast, esophageal, brain, renal, lung, cutaneous, colorectal, endometrial, ovarian, and cervical.

### In human and in vitro studies

The isoforms GLUT 1 and 3 have been reported to be associated with poor survival.^42^, ^43^

Moreover it has recently been shown that GLUT‐3 as well as GLUT‐1 are hypoxia‐responsive in neural stem cells.^43^, ^44^

The expression of GLUT‐5 has been reported to be upregulated during hypoxia exposure to 1% O_2_ tension leading to a 3.3‐fold increase in the uptake of 2‐deoxyglocuse by adipocytes.

Increased glucose transport in malignant cells has also been associated with increased expression of glucose transporters, with overexpression of GLUT1 and/or GLUT3.^45^

The GLUT family is divided into three classes based on their sequence homology. They can be stimulated by different molecules and their expression is located both intracellularly and at the cell surface.

Class I comprises the well characterized GLUT‐1–GLUT‐4. Class II comprises the fructose transporter GLUT5 and three recently described proteins, GLUT7, GLUT9, and GLUT1 which have been implicated in metabolic disorders.^46^

Class III comprises newly described proteins, GLUT‐6, GLUT‐8, GLUT‐10, GLUT‐12.

Each of the glucose transporter proteins display different affinities for glucose and other hexoses such as fructose, galactose, mannose, glucosamine. GLUT1 gene is responsible for basal glucose uptake and is expressed in virtually all tissues under normal conditions^47^ GLUT2 is encoded by the SLC2A2 gene, with a low affinity for glucose and high‐affinity for glucosamine.^48^, ^49^

It has been shown in Xenopus oocytes that GLUT1, GLUT3 have a low affinity transport capacity for galactose, mannose and fructose and a high affinity for glucose. Tumor cells prefer to convert glucose into lactate instead of utilizing the mitochondrial metabolism and the oxidative phosphorylation chain for energy production.^50^, ^51^

### In human studies

Earlier studies have demonstrated the presence of mRNA from different GLUTs in human tumors and a significant increase in the mRNA for GLUTl in gastrointestinal cancers associated with an increased cell glycolytic metabolism.^52^

GLUT‐1 is the only isoform which links with tumor grade and standard uptake value (SUV) index on FDG‐PET test.^53^

The overexpression of GLUT‐1 has been found to be associated with fluorodeoxyglucose (FDG) uptake, notably in pancreatic carcinoma.^54^ Studies carried out on culture cells showed enhanced GLUT‐1 expression along with glycolytic enzymes in the presence of oncogenes. The other GLUT isoforms including GLUT‐2, GLUT‐3, GLUT‐4 did not change their expression comparing healthy tissue with tumor. An activation and at the same time a decreased degradation were found suggesting a potential prognostic and predictive role.

Another cancer in which GLUT‐1 expression was found to increase concurrently with high FDG uptake was breast cancer.^55^ GLUT‐1 was expressed on the cell membrane and cytoplasm, but unlike GLUT‐2, is able to discriminate between benign and malignant disease.

Among various types of tumor, neck and head tumor often express both isoforms GLUT‐1 and GLUT‐3.^56^

Mitogens, oncogenes, and growth factors are all able to stimulate glucose metabolism and glucose transporters especially in tumors characterized by a high cell growth rate.^57^, ^58^ The glucose transport rate influences tumor growth by acting on cell differentiation, transformation and mitosis. Different tissues such as liver, brain, adipocytes, and small bowel present high mRNA expression of different isoforms.^59^

The induction of glucose transporters is tightly linked with the induction of hypoxia‐inducible factor 1‐alpha (HIF‐1α), a factor able, in turn, to increase tumor metabolism, stimulate vascular angiogenesis and mitosis associated factors.^60^

A GLUT‐1 associated enzyme, the phosphofructo‐2‐kinase contains binding sites for HIF in hypoxia condition, thus confirming the tight relationship between these two conditions of high glycolytic metabolism and hypoxia.^61^

**Figure 2:**
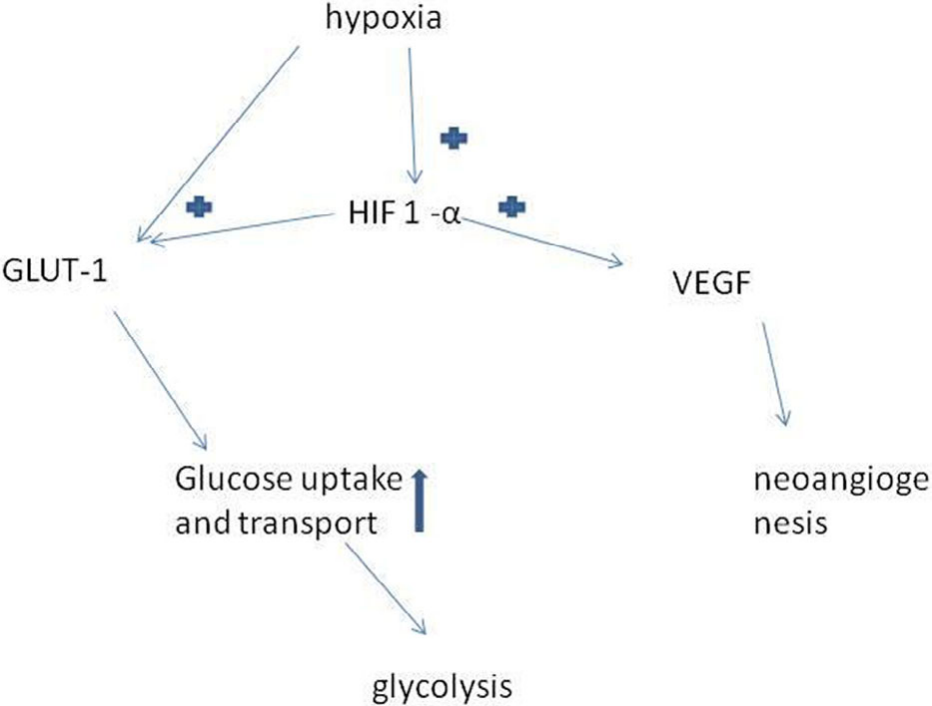
Effects of GLUT‐1 in relation to hypoxia. Hypoxia condition is a tissue oxygen tension of about 1% which stimulates tumor cells to produce HIF‐1α which in turn induces both the production of GLUT‐1 and VEGF; the first increase glucose uptake and cell metabolism whilst the second favors neoangiogenesis.

Furthermore, glucose deprivation favors the development of *KRAS* mutations in cancer which is an oncogene often associated with aggressive nonresponsive tumors.^62^ A condition of wild‐type for *KRAS* gene is associated with low‐glucose intake leading to reduced cell survival. *KRAS* mutation occurs in several malignancies such as colorectal, pancreatic and lung cancer. Clones with mutant *KRAS* present with an upregulation of GLUT‐1 gene and are associated with a poor patient prognosis. Table 2 represents the main factors activated by GLUT‐1 with its relative effects and Fig 2 shows the main pathways.

**Table 2:** Effects of GLUT‐1

Factors activated	Effects
HIF 1‐α	Tumor progression, neoangiogenesis
	cancer metabolism
AMPK phosphorylation	Altered tumor cell metabolism
Cyclin E2	Cell cycle progression G0‐G1
mTOR	Proteins synthesis, cell growth
*KRAS* mutation	Carcinogenesis, chemoresistance
PI3K	Increased glucose uptake and cancer metabolism
cMYC	Activation of glycolytic enzymes
EGFR expression	Tumor progression
FAK phosphorylation	Cancer cell migration

## GLUT 1 in lung cancer

Lung cancer is the leading cause of cancer death and different markers involved in cancer activation pathways are recognized to have a prognostic significance.^63^

### Preclinical studies

Indeed, transcription factors, oncogenes, cell cycle related genes participate in the process of tumor initiation and invasiveness, such as p21, Bcl‐2, p53 and cyclin D.

Adding GLUT‐1 RNA to lung cancer cell line A549 leads to a decrease of the expression of cyclins CDK 2 and 4 and conversely to an increased expression of p53 and 130 meaning that GLUT could affect cell cycle regulation.^64^ Moreover GLUT‐1 is able to increase the expression of adhesion molecules such as integrin β1 and focal adhesion kinase (FAK) favoring cancer cell migration.

GLUT‐1 is a prognostic factor that affects tumor aggressiveness and is used as a marker of staging in novel diagnostic techniques.^65^

### In human studies

In resected lung cancer, GLUT expression has been reported to be correlated with factors such as Ki‐67 and *KRAS* mutation and did not correlate with p53 or VEGF in untreated primary lung cancer.^49^, ^66^, ^67^ The VEGF and Ki‐67 have a prognostic factor in surgically‐treated lung cancer.^49^GLUT proteins not only play a role in tumor cell metabolism but also may have an effect on oncogenic factors such as *KRAS* mutation which has been found to be associated with the overexpression of GLUT‐1 in lung adenocarcinoma.

GLUT‐1 protein has been shown to actively interact with the immune system, and notably impaired GLUT expression seems to be a consequence of CD3/CD28 stimulation of T cells in secondary lung cancer pleural effusion.^67^ An altered glucose uptake has been linked with an impaired GLUT expression upon stimulation of T memory cells in pleural fluid. In hypoxia condition CD8+ T cell subsets from the nonmalignant group have an increased GLUT‐1 level in comparison with normoxia. Another aspect is the prognostic role of GLUT in NSCLC which is indicative of a poor outcome.^68^

The action of GLUT is strongly activated in cases of cancer tissue hypoxia^68^, ^69^ and could be overcome by wild‐type p53 oncosuppressor activity.^69^

A higher GLUT expression has been found in lung cancer metastatic sites than in primary sites in clinical studies indicating a relationship with fast growth and tumor spread.^50^, ^70^

Notably GLUT‐1, GLUT‐3 and GLUT‐5 expression levels are significantly higher in liver metastatic lung cancer compared with primary lung cancer and normal lung tissues.^70^

Other pathways are activated by GLUT‐1 in the mechanism of tumor growth and diffusion, and it is indeed able to influence the expression of matrix metalloproteinases (MMP) in hypoxia condition.^70^ The latter are proteinases that play a pivotal role in tissue remodeling and inflammatory processes and favoring carcinogenesis.

### In vitro and human studies

Concerning the relationship with chemotherapy, GLUT‐1 expression and glucose uptake are increased in gefitinib‐resistant NSCLC cells. Indeed, gefitinib treatment further decreased the number of viable cells and increased the probability of cell death in GLUT‐1 knockdown cells.^71^

Glucose uptake was increased in resistant NSCLC cells after gefitinib treatment suggesting that glucose intake and transport may be modulated by the action of anti‐EGFR drugs.

Studies curried out on lung cancer cell lines with western blot analysis revealed further important findings.

The GLUT expression may be reduced by a molecule called apigenin in lung cancer as revealed in studies carried out in animal models or in vitro.^72^ It is a molecule able to suppress glucose intake in lung cancer cells leading to cell apoptosis.

An overexpression of GLUT‐1 has been associated with major glucose uptake and SUV value together with tumor size^73^, ^74^ Concurrent altered regulation of GLUT‐related glucose uptake and vascular endothelial growth factor (VEGF) has been found in studies carried out on cell cultures in hypoxia condition. Indeed, an upregulation of VEGF and GLUT mRNA has been observed.^75^, ^76^

GLUT amplification has been associated with another factor such as sialyl Lewis x (sLe^x^) implicated in tumor growth, intercell crosstalk and cell proliferation^77^ determining a high glucose metabolism inside the cell and high cell proliferation rate.^77^, ^78^

An accelerated tumor cell metabolism has also been reported combined with EGFR‐tyrosine kinase which favors the maintaining of aerobic glycolysis in lung adenocarcinoma cells called the Warburg effect.^79^

By contrast, EGFR inhibitors (TKIs) decrease lactate production and glucose consumption, decreasing glycolytic metabolites. In lung cancer with activating *EGFR* mutations several genes are upregulated by oncogenes and are correlated with metabolism, such as GLUT‐1, hexokinase 2 (HK2), piruvate kinase and lactate dehydrogenase (LDH).^79^, ^80^, ^81^, ^82^

Other factors involved in cell metabolism favor aerobic glycolysis which is affected by tyrosine residues and can be mediated by phosphatidylinositol 3‐kinase (PI3K)/mammalian target of rapamycin (mTOR).^80^, ^81^

A new therapeutic agent called WZB117 able to inhibit glucose transport in human red blood cells expressing GLUT‐1 leads to a decrease in GLUT‐1 protein and glycolytic enzyme levels. These changes are followed by a decline in cyclin E2 and phosphorylated RB leading to cell cycle G0 arrest and necrosis.^83^

Among the enzymatic pathways induced, GLUT‐1 stimulates the protein pyruvate kinase muscle isozyme M2 (PKM2) which is involved in cancer cells with high metabolism rate.^84^ PKM2 is in turn responsible for mTOR inducing the Warburg effect.^84^, ^85^, ^86^ The Warburg effect can also be induced by p53 mutation.^86^, ^87^, ^88^ Eventually, there is an association of GLUT‐1 with the grade of lung tumor differentiation and staging, notably it is highly expressed with hexokinase in lung cancer.^89^, ^90^ In contrast, a downregulation of GLUT activity is carried out by c‐myc and other oncoproteins.^91^, ^92^ Furthermore a high expression of the above is correlated with an AMP‐activated protein kinase (AMPK) decreased function and an increase of cyclin E2 action favoring tumor spread. Epigenetic alterations are eventually inducers of GLUTs by causing histone post‐translational modifications as indicated in the results of studies carried out in lung cancer cell lines.^93^, ^94^

## Discussion

There are various signaling pathways which induce carcinogenesis and tumor growth. Lung cancer is the leading cause of cancer death and several causes are recognized among which are environmental factors and cigarette smoking.

Since it is a fast‐growing tumor characterized by high morbidity and mortality, several genes, transcriptional factors and metabolic pathways are involved in its development. Both metabolic factors and molecules involved in the cell cycle are crucial in the stepwise cancer evolution.

The two key factors considered in the present review are able to interact with pro‐ and anti‐apoptotic molecules and can also interact with growth and transcriptional factors.^95^

The association of glucose transporters with hypoxia inducible factor expressed in tissue with oxygen tension of 1%, in human lung cancer suggests that fast tumor growth requires an anaerobic effective glycolysis. We also know that glycolysis may occur in both normoxia and hypoxia conditions leading to ATP production, the so‐called Warburg effect.

Exogenous factors may affect tumor development such as cigarette smoke which is by far the main avoidable cause of lung cancer, the compounds of which could on their own stimulate the production of transcriptional molecules such as HIF‐1. In the current review, we focused on the main role of two important factors such as GLUT and p16 acting in opposite manner.

Nicotine derivatives are mainly able to induce pathways involving HIF‐1 and GLUT‐1 by acting on nicotinic acetylcholine receptors.^96^

HIF‐1 in turn is by itself able to activate downstream signals leading to angiogenesis.

Nicotine derivatives potentially can inactivate p16 by inducing cyclin D1 to stimulate G1 phase cell cycle progression.^97^

On the other hand, p16‐INK is in turn overexpressed in malignancies and is associated with HPV infection which by its envelope oncoproteins targets the pRb gene stimulating downstream genes involved in G1/S transition. The loss of pRb leads to p16‐INK upregulation.^98^, ^99^

Among the HPV‐derived proteins, E6 and E7 are often associated with high p16 expression and its dysregulation is favored by cigarette tobacco compounds such as benzopyrene inducing downstream activation of cyclin dependent kinase 1.^100^

Nicotine‐derived nitrosamines are carcinogenic factors that cause p16 inactivation hindering cisplatin proapoptosis action by Bcl‐2 gene induction and playing a possible role in determining chemotherapy failure.^4^, ^101^

The growth and spread of malignant cancer and notably lung cancer is the result of a close relationship between protective factors such as p16 and tumor stimulating factors.

Cell cycle, apoptosis and cell metabolism are all mechanisms subject to regulation. A fast metabolism needs fast glucose uptake and intake occurring in hypoxia condition and it is followed by VEGF‐induced neoangiogenesis and activation of transcriptional factors such as STAT.^102^

Hypoxia in turn activates SLC2A1 and A3 transcription which encodes the high affinity glucose transporters 1 and 3. At the same time it activates enzymes such as hexokinases which are involved in glycolytic pathways so that glucose is converted into pyruvate and downstream to lactic acid by the action of the enzyme LDH, ending up in the tricarboxylic acid cycle.^103^

These findings confirm that there is a relationship between GLUT‐1 and p16, and therefore they could both be useful in determining lung cancer prognosis.

Both immunohistochemical and western blot analyses showed high expression in the nucleus which correlated with a worse prognosis in patients.^104^

There is also evidence that GLUT‐1 was potentially useful in distinguishing benign from malignant mesothelial tumor apart from other molecules such as epithelial membrane antigen (EMA) and p53.^105^

In conclusion, our findings suggest that different types of environmental, genetic and metabolic factors are involved in lung tumor development and spread. A high metabolism rate was observed in tumor cells and this was associated with a high glucose intake, high aerobic and anaerobic glycolysis leading to the Warburg effect. GLUT plays a key role in cancer metabolism growth and progression. Among the isoforms only GLUT‐1 has been correlated with malignant tumor spread. Several studies in vitro and in humans have highlighted the mechanism of influence of that molecule on cell cycle and tumor spread which is associated with chemoresistance and a worse prognosis of lung cancer by stimulating cancer cell metabolism and growth, inhibiting apoptosis and favoring angiogenesis.

By contrast, p16 has an opposite action, favoring cell apoptosis and binding to cyclins CDK 4 and 6 determining, eventually, the arrest of cell cycle in G0 phase inducing tumor cell apoptosis. Therefore, both p16 and GLUT1 are overexpressed in malignant lung cancer and are potential prognostic factors, available for new target therapeutics.

## Disclosure

The authors declare that there are no conflicts of interest.

## References

[1] Mao Y , Yang H , Krasna M . Epidemiology of lung cancer. Surg Oncol Clin N Am 2016; 25: 439–45. 27261907 10.1016/j.soc.2016.02.001

[2] Osmani L , Askin F , Gabrielson E , Li QK . Current WHO guidelines and the critical role of immunohistochemical markers in the subclassification of non‐small cell lung carcinoma [NSCLC]: Moving from targeted therapy to immunotherapy. Semin Cancer Biol 2018; 52: 103–9. 29183778 10.1016/j.semcancer.2017.11.019 PMC5970946

[3] Pezzuto A , Stellato M , Catania G *et al* Short‐term benefit of smoking cessation along with glycopirronium on lung function and respiratory symptoms in mild COPD patients: A retrospective study. J Breath Res 2018; 12: 046007 10.1088/1752-7163/aad0a8. 29967309

[4] Condoluci A , Mazzara C , Zoccoli A , Pezzuto A , Tonini G . Impact of smoking on lung cancer treatment effectiveness: A review. Future Oncol 2016; 12 (189)): 2149–61. 10.2217/fon-2015-0055. 27424719

[5] Pezzuto A , Citarella F , Croghan I , Tonini G . The effects of cigarette smoking extracts on cell cycle and tumor spread: Novel evidence. Future Sci OA 2019; 5: FSO394. 31205749 10.2144/fsoa-2019-0017 PMC6556819

[6] Almetwally AA , Bin‐Jumah M , Allam AA . Ambient air pollution and its influence on human health and welfare: An overview. Environ Sci Pollut Res Int 2020; 27: 24815–30. 10.1007/s11356-020-09042-2. 32363462

[7] Takano N , Ariyasu R , Koyama J *et al* Improvement in the survival of patients with stage IV non‐small‐cell lung cancer: Experience in a single institutional 1995‐2017. Lung Cancer 2019; 131: 69–77. 31027701 10.1016/j.lungcan.2019.03.008

[8] Duma N , Santana‐Davila R , Molina JR . Non‐small cell lung cancer: Epidemiology, screening, diagnosis, and treatment. Mayo Clin Proc 2019; 94: 1623–40. 31378236 10.1016/j.mayocp.2019.01.013

[9] Maci E , Comito F , Frezza AM , Tonini G , Pezzuto A . Lung nodule and functional changes in smokers after smoking cessation short‐term treatment. Cancer Invest 2014; 32: 388–93. 24941266 10.3109/07357907.2014.919308

[10] Macheda M , Rogers S , Best JD . Molecular and cellular regulation of glucose transporter (GLUT) proteins in cancer. J Cell Physiol 2005; 202: 654–62. 15389572 10.1002/jcp.20166

[11] Li J , Poi MJ , Tsai MD . Regulatory mechanisms of tumor suppressor P16 INK4A and their relevance to cancer. Biochemistry 2011; 50: 5566–82. 21619050 10.1021/bi200642e PMC3127263

[12] Dong L , Zhang L , Hu SY *et al* Risk stratification of HPV 16 DNA methylation combined with E6 oncoprotein in cervical cancer screening: A 10‐year prospective cohort study. Clin Epigenetics 2020; 12 (1): 62 10.1186/s13148-020-00853-1. 32381054 PMC7204324

[13] Lassen P , Eriksen JG , Hamilton‐Dutoit S , Tramm T , Alsner J , Overgaard J . Danish head and neck cancer group (DAHANCA). HPV‐associated p16‐expression and response to hypoxic modification of radiotherapy in head and neck cancer. Radiother Oncol 2010; 94 (1): 30–5. 10.1016/j.radonc.2009.10.008. 19910068

[14] Reimers N , Kasper HU , Weissenborn SJ *et al* Combined analysis of HPV‐DNA, p16 and EGFR expression to predict prognosis in oropharyngeal cancer. Int J Cancer 2007; 120 (8): 1731–8. 17236202 10.1002/ijc.22355

[15] Crusius K , Auvinen E , Steuer B , Gaissert H , Alonso A . The human papilloma virus type 16 E5‐protein modulates ligand‐dependent activation of the EGF receptor family in the human epithelial cell line HaCaT. Exp Cell Res 1998; 241: 76–83. 9633515 10.1006/excr.1998.4024

[16] Wittekindt C , Gultekin E , Weissenborn SJ , Dienes HP , Pfister HJ , Klussmann JP . Expression of p16 protein is associated with human papilloma virus status in tonsillar carcinomas and has implications on survival. Adv Otorhinolaryngol 2005; 62: 72–80. 15608419 10.1159/000082474

[17] Grønhøj Larsen C , Gyldenløve M , Jensen DH *et al* Correlation between human papillomavirus and p16 overexpression in oropharyngeal tumours: A systematic review. Br J Cancer 2014; 110 (6): 1587–94. 10.1038/bjc.2014.42. 24518594 PMC3960616

[18] Lukic A , Sbenaglia G , Carico E *et al* Prediction of clinical outcome using p16INK4a immunocytochemical expression in low‐grade squamous intraepithelial lesions and high‐risk HPV‐positive atypical squamous cells of undetermined significance in patients with and without colposcopic evident cervical disease. Exp Ther Med 2011; 2: 853–8. 22977588 10.3892/etm.2011.316 PMC3440826

[19] Tam KW , Zhang W , Soh J *et al* CDKN2A/p16 inactivation mechanisms and their relationship to smoke exposure and molecular features in non‐small‐cell lung cancer. J Thorac Oncol 2013; 8: 1378–88. 24077454 10.1097/JTO.0b013e3182a46c0c PMC3951422

[20] CL W , Roz L , McKown S *et al* DNA studies underestimate the major role of CDKN2A inactivation in oral and oropharyngeal squamous cell carcinomas. Genes Chromosomes Cancer 1999; 25: 16–25. 10221335

[21] Zhou Y , Höti N , Ao M *et al* Expression of p16 and p53 in non‐small‐cell lung cancer: Clinicopathological correlation and potential prognostic impact. Biomark Med 2019; 13: 761–71. 31157548 10.2217/bmm-2018-0441 PMC8173521

[22] Roussel MF . The INK4 family of cell cycle inhibitors in cancer. Oncogene 1999; 18: 5311–7. 10498883 10.1038/sj.onc.1202998

[23] Romagosa C , Simonetti S , López‐Vicente L *et al* p16(Ink4a) overexpression in cancer: A tumor suppressor gene associated with senescence and high‐grade tumors. Oncogene 2011; 30 (18): 2087–97. 10.1038/onc.2010.614. 21297668

[24] Lin YC , Diccianni MB , Kim Y *et al* Human p16γ, a novel transcriptional variant of p16INK4A, coexpresses with p16INK4A in cancer cells and inhibits cell‐cycle progression. Oncogene 2007; 26: 7017–27. 17486064 10.1038/sj.onc.1210507

[25] Fåhraeus R , Paramio JM , Ball KL , Laín S , Lane DP . Inhibition of pRb phosphorylation and cell cycle progression by a 20‐residue peptide derived from p16CDKN2/INK4A. Curr Biol 1996; 6: 84–91. 8805225 10.1016/s0960-9822(02)00425-6

[26] Tominaga M , Sueoka N , Irie K . Detection and discrimination of preneoplastic and early stages of lung adenocarcinoma using heterogeneous nuclear ribonucleoproteins hnRNP B1 combined with the cell cycle‐related markers p16, cyclin D1, and Ki‐67. Lung Cancer 2003; 40 (1): 45–53. 12660006 10.1016/s0169-5002(02)00529-9

[27] Arifin M , Tanimoto K , Putra AC , Hiyama E , Nishiyama M , Hiyama K . Carcinogenesis and cellular immortalization without persistent inactivation of p16/Rb pathway in lung cancer. Int J Oncol 2010; 36 (5): 1217–27. 20372796 10.3892/ijo_00000605

[28] Al‐Mohanna MA , Manogaran PS , Al‐Mukhalafi ZK , Al‐hussein A , Aboussekhra A . The tumor suppressor p16 NK4a gene is a regulator of apoptosis induced by ultraviolet light and cisplatin. Oncogene 2004; 23: 201–12. 14712225 10.1038/sj.onc.1206927

[29] Inamura K . Clinicopathological characteristics and mutations driving development of early lung adenocarcinoma: Tumor initiation and progression. Int J Mol Sci 2018; 19 (4): E1259 10.3390/ijms19041259. 29690599 PMC5979290

[30] Witkiewicz AK , Knudsen KE , Dicker AP , Knudsen ES . The meaning of p16ink4a expression in tumors functional significance, clinical associations and future developments. Cell Cycle 2011; 15: 2497–503. 10.4161/cc.10.15.16776 PMC3685613 21775818

[31] Pezzuto A , Cappuzzo F , D'Arcangelo M *et al* Prognostic value of p16 protein in patients with surgically treated non‐small cell lung cancer; relationship with Ki‐67 and PD‐L1. Anticancer Res 2020; 40: 983–90. 32014943 10.21873/anticanres.14032

[32] Younes M , Lechago LV , Somoano JR , Mosharaf M , Lechago J . Wide expression of the human erythrocyte glucose transporter Glut1 in human cancers. Cancer Res 1996; 56: 1164–7. 8640778

[33] Tachibana M , Saito M , Kobayashi J , Isono T , Yatabe Y , Tsutsumi Y . Distal‐type bronchiolar adenoma of the lung expressing p16INK4a ‐ morphologic, immunohistochemical, ultrastructural and genomic analysis ‐ report of a case and review of the literature. Pathol Int 2020; 70 (3): 179–85. 32030846 10.1111/pin.12904 PMC7079048

[34] Kashiwabara K , Oyama T , Sano T , Fukuda T , Nakajima T . Correlation between methylation status of the p16/CDKN2 gene and the expression of p16 and Rb proteins in primary non‐small cell lung cancers. Int J Cancer 1998; 79 (3): 215–20. 9645340 10.1002/(sici)1097-0215(19980619)79:3<215::aid-ijc1>3.0.co;2-s

[35] Tuo L , Sha S , Huayu Z , Du K . p16INK4a gene promotor methylation as a biomarker for the diagnosis of non‐small cell lung cancer: An updated meta‐analysis. Thorac Cancer 2018; 9: 1032–40. 29927090 10.1111/1759-7714.12783 PMC6068431

[36] Gu J , Wen Y , Zhu S *et al* Association between P(16INK4a) promoter methylation and non‐small cell lung cancer: A meta‐analysis. PLOS One 2013; 8: e60107. 23577085 10.1371/journal.pone.0060107 PMC3618325

[37] Kersting M , Friedl C , Kraus A , Behn M , Pankow W , Schuermann M . Differential frequencies of p16[INK4a] promoter hypermethylation, p53 mutation, and K‐ras mutation in exfoliative material mark the development of lung cancer in symptomatic chronic smokers. J Clin Oncol 2000; 18: 3221–9. 10986054 10.1200/JCO.2000.18.18.3221

[38] Bearzatto A , Conte D , Frattini M *et al* p16(INK4A) hypermethylation detected by fluorescent methylation‐specific PCR in plasmas from non‐small cell lung cancer. Clin Cancer Res 2002; 8: 3782–7. 12473590

[39] Zhou Y , Höti N , Ao M *et al* Expression of p16 and p53 in nonsmall‐cell lung cancer: Clinicopathological correlation and potential prognostic impact. Biomark Med 2019; 13: 761–71. 31157548 10.2217/bmm-2018-0441 PMC8173521

[40] Miyoshi R , Menju T , Yoshizawa A , Date H . Expression of p16Ink4a in mixed squamous cell and glandular papilloma of the lung. Pathol Int 2017; 67: 306–10. 28470939 10.1111/pin.12531

[41] Wu M , Orta L , Gil J , Li G , Hu A , Burstein DE . Immunohistochemical detection of XIAP and p63 in adenomatous hyperplasia, atypical adenomatous hyperplasia, bronchioloalveolar carcinoma and well differentiated adenocarcinoma. Mod Pathol 2008; 21: 553–8. 18432259 10.1038/modpathol.2008.5

[42] Barron CC , Bilan PJ , Tsakiridis T , Tsiani E . Facilitative glucose transporters: Implications for cancer detection, prognosis and treatment. Metabolism 2016; 65: 124–39. 26773935 10.1016/j.metabol.2015.10.007

[43] Wood IS , Wang B , Lorente‐Cebrián S , Trayhurn P . Hypoxia increases expression of selective facilitative glucose transporters (GLUT) and 2‐deoxy‐D‐glucose uptake in human adipocytes. Biochem Biophys Res Commun 2007; 361: 468–73. 17658463 10.1016/j.bbrc.2007.07.032 PMC2211375

[44] Fukumoto H , Seino S , Imura H *et al* Sequence, tissue distribution, and chromosomal localization of mRNA encoding a human glucose transporter‐like protein. Proc Natl Acad Sci U S A 1988; 85: 5434–3438. 3399500 10.1073/pnas.85.15.5434 PMC281771

[45] Wieczorke R , Dlugai S , Krampe S , Boles E . Characterisation of mammalian GLUT glucose transporters in a heterologous yeast expression system. Cell Physiol Biochem 2003; 13: 123–34. 12876383 10.1159/000071863

[46] Ziegler GC , Almos P , McNeill RV , Jansch C , Lesch KP . Cellular effects and clinical implications of SLC2A3 copy number variation. J Cell Physiol 2020; 1: 1–16. 10.1002/jcp.29753. 32372501

[47] Zhou L , Li S , Liu L , Zhou Q , Yuan Y , Xin L . Recombinant methioninase regulates PI3K/Akt/Glut‐1 pathway and inhibits aerobic glycolysis to promote apoptosis of gastric cancer cells. Nan Fang Yi Ke Da Xue Xue Bao 2020; 40: 27–33. 32376548 10.12122/j.issn.1673-4254.2020.01.05 PMC7040764

[48] Liu Y , Li YM , Tian RF *et al* The expression and significance of HIF‐1alpha and GLUT‐3 in glioma. Brain Res 2009; 1304: 149–54. 19782666 10.1016/j.brainres.2009.09.083

[49] Minami K , Saito Y , Imamura H . Prognostic significance of p53, Ki‐67, VEGF and Glut‐1 in resected stage I adenocarcinoma of the lung. Lung Cancer 2002; 38: 51–7. 12367793 10.1016/s0169-5002(02)00108-3

[50] Brown RS , Leung JY , Kison PV , Zasadny KR , Flint A , Wahl RL . Glucose transporters and FDG uptake in untreated primary human non‐small cell lung cancer. J Nucl Med 1999; 40: 556–65. 10210213

[51] Pezzuto A , Carico E . Role of HIF‐1 in cancer progression: Novel insights. A Review. Curr Mol Med 2018; 18: 343–51. 30411685 10.2174/1566524018666181109121849

[52] Meyer HJ , Wienke A , Surov A . Associations between GLUT expression and SUV values derived from FDG‐PET in different tumors‐a systematic review and meta analysis. PLOS One 2019; 14: e0217781. 31206524 10.1371/journal.pone.0217781 PMC6576787

[53] Higashi T , Tamaki N , Honda T *et al* Expression of glucose transporters in human pancreatic tumors compared with increased FDG accumulation in PET study. J Nucl Med 1997; 38 (9): 1337–44. 9293783

[54] Reske SN , Grillenberger KG , Glatting G *et al* Overexpression of glucose transporter 1 and increased FDG uptake in pancreatic carcinoma. J Nucl Med 1997; 38: 1344–8. 9293784

[55] Brown RS , Wahl RL . Overexpression of Glut‐1 glucose transporter in human breast cancer. An immunohistochemical study. Cancer 1993; 72: 2979–85. 8221565 10.1002/1097-0142(19931115)72:10<2979::aid-cncr2820721020>3.0.co;2-x

[56] Mellanen P , Minn H , Grenman R , Harkonen P . Expression of glucose transporters in head‐and‐neck tumors. Int J Cancer 1994; 56: 622–9. 8314336 10.1002/ijc.2910560503

[57] Merrall NW , Plevin R . Growth factors, mitogens, oncogenes and the regulation of glucose transport. Cell Signal 1993; 5: 667–75. 8130071 10.1016/0898-6568(93)90028-k

[58] Mueckler M . Facilitative glucose transporters. Eur J Biochem 1994; 219 (3): 713–25. 8112322 10.1111/j.1432-1033.1994.tb18550.x

[59] Yamamoto T , Seino Y , Fukumoto H *et al* Over‐expression of facilitative glucose transpo.ter genes in human cancer. Biochem Biophys Res Commun 1990; 170: 223–30. 2372287 10.1016/0006-291x(90)91263-r

[60] Hayashi M , Sakata M , Takeda T *et al* Induction of glucose transporter 1 expression through hypoxia‐inducible factor 1alpha under hypoxic conditions in trophoblast‐derived cells. J Endocrinol 2004; 183: 145–54. 15525582 10.1677/joe.1.05599

[61] Obach M , Navarro‐Sabate A , Caro J *et al* 6‐Phosphofructo‐2‐kinase [pfkfb3] gene promoter contains hypoxia‐inducible factor‐1 binding sites necessary for transactivation in response to hypoxia. J Biol Chem 2004; 279: 53562–70. 15466858 10.1074/jbc.M406096200

[62] Yun J , Rago C , Cheong I *et al* Glucose deprivation contributes to the development of KRAS pathway mutations in tumor cells. Science 2009; 325: 1555–9. 19661383 10.1126/science.1174229 PMC2820374

[63] Niklinski J , Niklinska W , Laudanski J *et al* Prognostic molecular markers in non‐small cell lung cancer. Lung Cancer 2001; S53 (8): 34. 10.1016/s0169-5002(01)00345-2 11720742

[64] Zhao H , Sun J , Shao J . Glucose transporter 1 promotes the malignant phenotype of non‐small cell lung cancer through integrin β1/Src/FAK signaling. J Cancer 2019; 10: 4989–97. 31598171 10.7150/jca.30772 PMC6775508

[65] Brown RS , Leung JY , Kison PV , Zasadny KR , Flint A , Wahl RL . Glucose transporters and FDG uptake in untreated primary human non‐small cell lung cancer. J Nucl Med 1999; 40 (4): 556–65. 10210213

[66] Sasaki H , Shitara M , Yokota K . Overexpression of GLUT1 correlates with Kras mutations in lung carcinomas. Mol Med Rep 2012; 5: 599–602. 22200795 10.3892/mmr.2011.736

[67] Prado‐Garcia H , Romero‐Garcia S , Castro‐Flores DA , Rumbo‐Nava U . Deficient glucose uptake is linked to impaired Glut1 expression upon CD3/CD28 stimulation in memory T cells from pleural effusions secondary to lung cancer. Scand J Immunol 2019; 90: e12802. 31269269 10.1111/sji.12802

[68] Tan Z , Yang C , Zhang X , Zheng P , Shen W . Expression of glucose transporter 1 and prognosis in non‐small cell lung cancer: A pooled analysis of 1665 patients. Oncotarget 2017; 8: 60954–61. 28977837 10.18632/oncotarget.17604 PMC5617397

[69] Schwartzenberg‐Bar‐Yoseph F , Armoni M , Karnieli E . The tumor suppressor p53 down‐regulates glucose transporters GLUT1 and GLUT4 gene expression. Cancer Res 2004; 64: 2627–33. 15059920 10.1158/0008-5472.can-03-0846

[70] Kurata T , Oguri T , Isobe T , Ishioka S , Yamakido M . Differential expression of facilitative glucose transporter (GLUT) genes in primary lung cancers and their liver metastases. Jpn J Cancer Res 1999; 90 (11): 1238–43. 10622535 10.1111/j.1349-7006.1999.tb00702.x PMC5926010

[71] Suzuki S , Okada M , Takeda H *et al* Involvement of GLUT1‐mediated glucose transport and metabolism in gefitinib resistance of non‐small‐cell lung cancer cells. Oncotarget 2018; 9: 32667–79. 30220973 10.18632/oncotarget.25994 PMC6135698

[72] Lee Y , Lee G , Oh T *et al* Inhibition of glutamine utilization sensitizes lung cancer cells to apigenin‐induced apoptosis resulting from metabolic and oxidative stress. Int J Oncol 2015; 48: 399–408. 10.3892/ijo.2015.3243. 26573871

[73] Suzawa N , Ito M , Qiao S *et al* Assessment of factors influencing FDG uptake in non‐small cell lung cancer on PET/CT by investigating histological differences in expression of glucose transporters 1 and 3 and tumour size. Lung Cancer 2011; 72: 191–8. 20884076 10.1016/j.lungcan.2010.08.017

[74] Berghmans T , Dusart M , Paesmans M *et al* Primary tumor standardized uptake value (SUVmax) measured on fluorodeoxyglucose positron emission tomography (FDG‐PET) is of prognostic value for survival in non‐small cell lung cancer (NSCLC): A systematic review and meta‐analysis (MA) by the European Lung Cancer Working Party for the IASLC Lung Cancer Staging Project. J Thorac Oncol 2008; 3: 6–12. 18166834 10.1097/JTO.0b013e31815e6d6b

[75] Pedersen MW , Holm S , Lund EL , Hojgaard L , Kristjansen PE . Coregulation of glucose uptake and vascular endothelial growth factor (VEGF) in two small‐cell lung cancer (SCLC) sublines in vivo and in vitro. Neoplasia 2001; 3 (1): 80–7. 11326319 10.1038/sj.neo.7900133 PMC1505028

[76] Yamamoto T , Seino Y , Fukumoto H *et al* Over‐expression of facilitative glucose transporter genes in human cancer. Biochem Biophys Res Commun 1990; 170 (1): 223–30. 2372287 10.1016/0006-291x(90)91263-r

[77] Ogawa J , Inoue H , Koide S . Glucose‐transporter‐type‐I‐gene amplification correlates with sialyl‐Lewis‐X synthesis and proliferation in lung cancer. Int J Cancer 1997; 74: 189–92. 9133454 10.1002/(sici)1097-0215(19970422)74:2<189::aid-ijc9>3.0.co;2-v

[78] Gillies RJ , Robey I , Gatenby RA . Causes and consequences of increased glucose metabolism of cancers. J Nucl Med 2008; 49 (Suppl 2): S24–42. 10.2967/jnumed.107.047258 18523064

[79] Makinoshima H , Takita M , Matsumoto S *et al* Epidermal growth factor receptor (EGFR) signaling regulates global metabolic pathways in EGFR‐mutated lung adenocarcinoma. J Biol Chem 2014; 289: 20813–23. 10.1074/jbc.M114.575464. 24928511 PMC4110289

[80] Lunt SY , Van der Heiden MG . Aerobic glycolysis: Meeting the metabolic requirements of cell proliferation. Annu Rev Cell Dev Biol 2011; 27: 441–64. 21985671 10.1146/annurev-cellbio-092910-154237

[81] Makinoshima H , Takita M , Saruwatari K *et al* Signaling through the phosphatidylinositol 3‐kinase (PI3K)/mammalian target of rapamycin (mTOR) axis is responsible for aerobic glycolysis mediated by glucose transporter in epidermal growth factor receptor (EGFR)‐mutated lung adenocarcinoma. J Biol Chem 2015; 290: 17495–504. 10.1074/jbc.M115.660498. 26023239 PMC4498084

[82] Soga T . Cancer metabolism: Key players in metabolic reprogramming. Cancer Sci 2013; 104: 275–81. 23279446 10.1111/cas.12085 PMC7657261

[83] Hitosugi T , Kang S , Van der Heiden MG *et al* Tyrosine phosphorylation inhibits PKM2 to promote the Warburg effect and tumor growth. Sci Signal 2009; 2: ra73. 19920251 10.1126/scisignal.2000431 PMC2812789

[84] Camp ER , Summy J , Bauer TW , Liu W , Gallick GE , Ellis L . M. Mechanisms of resistance to therapies targeting the epidermal growth factor receptor. Clin Cancer Res 2005; 11: 397–405. 15671571

[85] Liu Y , Cao Y , Zhang W *et al* A small molecule inhibitor of glucose transporter 1 downregulates glycolysis, induces cell‐cycle arrest, and inhibits cancer cell growth in vitro and in vivo. Mol Cancer Ther 2012; 11: 1672–82. 10.1158/1535-7163.mct-12-0131. 22689530

[86] Wong N , Ojo D , Yan J , Tang D . PKM2 contributes to cancer metabolism. Cancer Lett 2014; 356: 184–91. 24508027 10.1016/j.canlet.2014.01.031

[87] Yang W , Lu Z . Nuclear PKM2 regulates the Warburg effect. Cell Cycle 2013; 12: 3154–8. 24013426 10.4161/cc.26182 PMC3865010

[88] Zhang C , Liu J , Liang Y *et al* Tumour‐associated mutant p53 drives the Warburg effect. Nat Commun 2013; 4: 2935. 24343302 10.1038/ncomms3935 PMC3969270

[89] Ito T , Noguchi Y , Satoh S , Hayashi H , Inayama Y , Kitamura H . Expression of facilitative glucose transporter isoforms in lung carcinomas: Its relation to histologic type, differentiation grade, and tumor stage. Mod Pathol 1998; 11: 437–43. 9619596

[90] Mamede M , Higashi T , Kitaichi M *et al* [^18^F]FDG uptake and PCNA, Glut‐1, and hexokinase‐II expressions in cancers and inflammatory lesions of the lung. Neoplasia 2005; 7 (4): 369–79. 15967114 10.1593/neo.04577 PMC1501150

[91] Merrall NW , Plevin R , Gould GW . Growth factors, mitogens, oncogenes, and the regulation of glucose transport. Cell Signal 1993; 5: 667–75. 8130071 10.1016/0898-6568(93)90028-k

[92] Cairns RA , Harris IS , Mok TW . Regulation of cancer cell metabolism. Nat Rev Cancer 2011; 11: 85–95. 21258394 10.1038/nrc2981

[93] Levine AJ , Puzio‐Kuter AM . The control of the metabolic switch in cancers by oncogenes and tumor suppressor genes. Science 2010; 330: 1340–4. 21127244 10.1126/science.1193494

[94] O'Byrne KJ , Baird AM , Kilmartin L , Leonard J , Sacevich C , Gray SG . Epigenetic regulation of glucose transporters in non‐small cell lung cancer. Cancers (Basel) 2011; 25 (2): 1550–65. 10.3390/cancers3021550 PMC3757377 24212773

[95] Ratschiller D , Heighway J , Gugger M *et al* Cyclin D1 overexpression in bronchial epithelia of patients with lung cancer is associated with smoking and predicts survival. J Clin Oncol 2003; 21 (11): 2085–93. 12775733 10.1200/JCO.2003.03.103

[96] Zhang Q , Tang X , Zhang ZF , Velikina R , Shi S , Le AD . Nicotine induces hypoxia inducible factor‐1 alpha expression in human lung cancer cells via nicotinic acetylcholine receptor‐mediated signaling pathways. Clin Cancer Res 2007; 13 (16): 4686–94. 17699846 10.1158/1078-0432.CCR-06-2898 PMC4166418

[97] Chu M , Guo J , Chen CY . Long‐term exposure to nicotine via RAS pathway induces cyclin D1 to stimulate G1 cell cycle transition. J Biol Chem 2005; 280 (8): 6369–79. 15574422 10.1074/jbc.M408947200

[98] Goto A , Li CP , Ota S *et al* Human papillomavirus infection in lung and esophageal cancers: Analysis of 485 Asian cases. J Med Virol 2011; 83: 1383–90. 21678442 10.1002/jmv.22150

[99] Narisawa SM , Kiyono T . Basic mechanisms of high risk human papillomavirus‐induced carcinogenesis: Roles of E6 and E7 proteins. Cancer Sci 2007; 98: 1505–11. 17645777 10.1111/j.1349-7006.2007.00546.x PMC11158331

[100] Alam S , Bowser BP , Conway MJ *et al* Downregulation of cdk2/1 kinase activity induces the synthesis of non infectious human papillomavirus type 31 b virions in organotypic tissues exposed to benzopyrene. J Urol 2010; 84: 4630–45. 10.1128/JVI.02431-09 PMC2863740 20181698

[101] Zeng F , Li YC , Chen G *et al* Nicotine inhibits cisplatin‐induced apoptosis in NCI‐H446 cells. Med Oncol 2012; 29: 364–73. 21267677 10.1007/s12032-010-9792-9

[102] Thorgeirsson TE , Geller F , Sulem P *et al* Variant associated with nicotine dependence, lung cancer and peripheral arterial disease. Nature 2008; 452: 638–42. 18385739 10.1038/nature06846 PMC4539558

[103] MG VH , Cantley LC , Thompson CB . Understanding the Warburg effect: The metabolic requirements of cell proliferation. Science 2009; 324: 1029–33. 19460998 10.1126/science.1160809 PMC2849637

[104] Groeger AM , Caputi M , Esposito V *et al* Independent prognostic role of p16 in lung cancer. J Thorac Cardiovasc Surg 1999; 118 (3): 529–35. 10469971 10.1016/s0022-5223(99)70192-3

[105] Hasteh F , Lin G , Weidner N , Michael CW . The use of immunohistochemistry to distinguish reactive mesothelial cells from malignant mesothelioma in cytologic effusions. Cancer 2010; 118: 90–6. 10.1002/cncy.20071 20209622

